# Antenatal depression programs cortisol stress reactivity in offspring through increased maternal inflammation and cortisol in pregnancy: The Psychiatry Research and Motherhood – Depression (PRAM-D) Study

**DOI:** 10.1016/j.psyneuen.2018.06.017

**Published:** 2018-12

**Authors:** S. Osborne, A. Biaggi, T.E. Chua, A. Du Preez, K. Hazelgrove, N. Nikkheslat, G. Previti, P.A. Zunszain, S. Conroy, C.M. Pariante

**Affiliations:** a King’s College London, Institute of Psychiatry, Psychology and Neuroscience, Department of Psychological Medicine, Section of Perinatal Psychiatry & Stress, Psychiatry and Immunology, The Maurice Wohl Clinical Neuroscience Institute, Cutcombe Road, London, SE5 9RX, UK; b King’s College London, Institute of Psychiatry, Psychology and Neuroscience, Department of Psychological Medicine, Section of Psychosis Studies, London, SE5 9AF, UK; c Department of Psychological Medicine, KK Women’s and Children’s Hospital, 100 Bukit Timah Road, Singapore 229899, Singapore; d King’s College London, Institute of Psychiatry, Psychology and Neuroscience, Department of Basic and Clinical Neuroscience, The Maurice Wohl Clinical Neuroscience Institute, Cutcombe Road, London, SE5 9RX, UK; e Department of Mental Health and Addiction, Via Risorgimento 57 42123, Reggio Emilia, Italy

**Keywords:** Antenatal depression, Inflammation, Cortisol, Infant, Pregnancy, Stress

## Abstract

**Introduction:**

Antenatal depression is associated with a broad range of suboptimal outcomes in offspring, although the underlying mechanisms are not yet understood. Animal studies propose inflammation and glucocorticoids as mediators of the developmental programming effect of prenatal stress on offspring stress responses, but studies in humans are not yet at this stage. Indeed, to date no single study has examined the effects of a rigorously defined, clinically significant Major Depressive Disorder (MDD) in pregnancy on maternal antenatal inflammatory biomarkers and hypothalamic-pituitary (HPA) axis, as well as on offspring HPA axis, behavior and developmental outcomes in the first postnatal year.

**Methods:**

A prospective longitudinal design was used in 106 women (49 cases vs. 57 healthy controls) to study the effect of MDD in pregnancy and associated antenatal biology (inflammatory and cortisol biomarkers), on offspring stress response (cortisol response to immunization, at 8 weeks and 12 months), early neurobehavior (Neonatal Behavioral Assessment Scale, NBAS, at day 6), and cognitive, language and motor development (Bayley Scales of Infant and Toddler Development at 12 months).

**Results:**

Compared with healthy controls, women with MDD in pregnancy had raised interleukin (IL) IL-6 (effect size (δ) = 0.53, p = 0.031), IL-10 (δ = 0.53, p = 0.043), tumor necrosis factor alpha (δ = 0.90, p = 0.003) and vascular endothelial growth factor (δ = 0.56, p = 0.008), together with raised diurnal cortisol secretion (δ = 0.89, p = 0.006), raised evening cortisol (δ = 0.64, p = 0.004), and blunted cortisol awakening response (δ = 0.70, p = 0.020), and an 8-day shorter length of gestation (δ = 0.70, p = 0.005). Furthermore, they had neonates with suboptimal neurobehavioral function in four out of five NBAS clusters measured (range of δ = 0.45–1.22 and p = 0.049–<0.001) and increased cortisol response to stress at one year of age (δ = 0.87, p < 0.001). Lastly, maternal inflammatory biomarkers and cortisol levels were correlated with infant stress response, suggesting a mechanistic link.

**Conclusion:**

This study confirms and extends the notion that depression in pregnancy is associated with altered offspring behavior and biological stress response, and demonstrates that changes in maternal antenatal stress-related biology are associated with these infant outcomes.

## Highlights

• Women with antenatal depression have higher stress-related biomarkers than controls.
• Women with antenatal depression have shorter length of gestation than controls.
• Neonates exposed to antenatal depression have suboptimal neurobehavioural function.
• 1-year-olds exposed to antenatal depression have increased cortisol stress-response.
• Maternal antenatal, and infant stress-related biomarkers are associated.

## 1 Introduction

In clinical studies, depression in pregnancy (antenatal depression) has been recognized as a key clinical risk factor for the transmission of abnormal mental health and behavior to the offspring generation, over and above the effects of disrupted maternal care due to postnatal depression (Davis et al., 2007; Pawlby et al., 2009; Gerardin et al., 2010; Hay et al., 2010; Pearson et al., 2013; Van Batenburg-Eddes et al., 2013). These effects of antenatal depression are wide-ranging, and include adverse effects on neonatal behavior (Zuckerman et al., 1990; Field et al., 2004a; Diego et al., 2005; Goodman et al., 2011; Pacheco and Figueiredo, 2012), infant development (Deave et al., 2008; Previti et al., 2014) and mental health later in childhood and adolescence (Davis et al., 2007; Pawlby et al., 2009; Hay et al., 2010; Pawlby et al., 2011; Pearson et al., 2013; Plant et al., 2013; Van Batenburg-Eddes et al., 2013; Previti et al., 2014; Stein et al., 2014; Plant et al., 2015, 2016; Plant et al., 2017). These specific antenatal effects point toward a direct biological effect of the in utero environment during depression on fetal brain development, resulting in the programming of an abnormal behavioral and biological stress response, eventually translating to increased psychopathology later in life; but this model is largely based on animal studies and limited human evidence (Glover, 2015). In humans, we still do not know the exact molecular and clinical mechanisms underlying the association between adverse in utero environment and offspring behavioral development.

Although mechanisms for developmental programming in humans are likely to be complex, it is postulated that, as in animals, exposure of the fetus to high levels of inflammatory biomarkers or glucocorticoids is a key mediator, resulting in subsequent effects on HPA axis, behavior, and cognitive function of the offspring (Seckl, 2001, 2004; Seckl and Holmes, 2007; Cottrell and Seckl, 2009; Reynolds, 2013; Bolton and Bilbo, 2014; Moisiadis and Matthews, 2014a, b; Debnath et al., 2015; Glover, 2015; Segovia et al., 2017). While current research lends support to this hypothesis, to date studies have largely used symptoms of depression in the general population as a paradigm for prenatal stress, rather than clinically significant, operationally defined, antenatal major depressive disorder (MDD). The advantage of examining MDD is that symptoms are at a disease level, thereby ensuring that stress is meaningful and produces clinically significant impairment. Indeed, studies of symptoms of depression in pregnancy have provided some evidence of the same altered inflammation and HPA axis activity found in depression outside of pregnancy (Pariante and Lightman, 2008; Miller and Raison, 2016; Pariante, 2017). For example, elevated levels of interleukin (IL) IL-1β, IL-6, tumor necrosis factor alpha (TNFα) and C-reactive protein (CRP) (Scrandis et al., 2008; Christian et al., 2009; Cassidy-Bushrow et al., 2012; Azar and Mercer, 2013; Haeri et al., 2013), and of corticotrophin releasing hormone (CRH) and cortisol (Rich-Edwards et al., 2008; O’Keane et al., 2011; O’Connor et al., 2013b), have all been described in association with antenatal symptoms of depression. However, no studies have examined both inflammation and HPA axis in the same depressed pregnant women. Accordingly, MDD is theoretically an ideal paradigm by which to study the molecular mechanisms underlying developmental programming.

Of note also is that developmental programming studies so far have only measured the associations between two variables at a time, limiting our understanding of the connections of antenatal depression with both offspring behavior and stress-related biology. For example, studies have found associations (i) between antenatal symptoms of depression and offspring HPA axis or inflammation (Field et al., 2004b; Brennan et al., 2008; Davis et al., 2011a; Vedhara et al., 2012; O’Donnell et al., 2013; Plant et al., 2016); or (ii) between antenatal HPA axis and offspring HPA axis (Gutteling et al., 2004, 2005; Davis et al., 2011b; O’Connor et al., 2013a); or (iii) between antenatal HPA axis and infant behaviors or cognitive and motor development (de Weerth et al., 2003; Huizink et al., 2003; Davis et al., 2007; Davis and Sandman, 2010); or (iv) between antenatal inflammation and behavioral outcomes in offspring (Graham et al., 2017). We are lacking a study combining a prospective assessment of: (i) clinically significant levels of antenatal stress in the form of MDD; (ii) antenatal maternal inflammation and HPA axis; (iii) gestational outcomes; (iv) offspring neurobehavioral function and development; and (v) offspring HPA axis. The current study, aptly called Psychiatry Research and Motherhood – Depression (PRAM-D) Study, addresses this existing gap in the literature by studying women with MDD in pregnancy (and healthy pregnant women) who are then followed-up, together with their offspring, up to one year postnatally.

## 2 Methodology

### 2.1 Design

In a prospective longitudinal observational study, we compared a cases group with a DSM-IV diagnosis of MDD in pregnancy (and their offspring) with a control group of healthy pregnant women (and their offspring) up to one year postnatally. Maternal socio-demographics, obstetric and physical risk factors, together with clinical status and inflammatory markers, were assessed at baseline (25 weeks gestation), and HPA axis at 32 weeks of pregnancy. Gestational age at birth and birth weight were recorded, and neonatal neurobehavioral function was assessed at 6-days postnatal. Infant cortisol reactivity (response to the pain stress of routine immunizations) and basal activity (morning and evening) was also assessed at 2- and 12-months postnatal. Finally, infant development was assessed at 12-months postnatal. Outcome measures were assessed blind to caseness. The study was approved by King’s College Hospital Research Ethics Committee, and all participants provided written informed consent.

### 2.2 Sample

The sample comprised 106 women recruited in the late second trimester of pregnancy (25 weeks gestation): 49 cases with MDD in pregnancy (referred to Maudsley Perinatal Psychiatry Services) and 57 healthy controls (attending routine antenatal ultrasound scan) all at King’s College Hospital. Of the 49 cases, 31 (63%) met DSM-IV criteria for MDD at 25 weeks gestation (baseline visit) and 18 (37%) were assessed to have met criteria for MDD in early pregnancy but not any longer by baseline. Of the total sample, 41 (84%) had a past history of MDD. Inclusion criteria were: women over 18 years of age with a singleton pregnancy; cases with a DSM-IV diagnosis of MDD in pregnancy; and controls without any current or past DSM-IV axis I diagnosis. Exclusion criteria were: uterine anomaly, known obstetric complications in the index pregnancy, severe or relevant chronic medical conditions, such as cardiovascular disease, metabolic or endocrine disorder, for example gestational diabetes and hypertension. Cases were excluded if presenting with any current DSM-IV diagnosis other than co-morbid anxiety disorder, if having a past history of psychosis or bipolar affective disorder, or if taking antidepressant medication at baseline.

As expected in a longitudinal study of an inner city group of people, subject retention reduced over time, and at 1 year postnatal only 87 mother-infant dyads (51 controls and 36 cases) were assessed. However, there was no statistically significant difference at any time point between the proportion of cases and controls remaining in the study; furthermore, there were no statistically significant differences in socio-demographic information at baseline between those who did and did not complete the 1 year assessments, either for cases or for controls (data not shown). However, subjects who did not complete the 1-year assessment had higher BDI and STAI scores at baseline and a greater proportion who had smoked in pregnancy (see below), which may have influenced some of the findings (see Discussion).

### 2.3 Clinical assessment

All subjects were assessed for current and past DSM-IV axis I disorders at baseline using the Structured Clinical Interview for DSM-IV (SCID I – CV) (First, 1996). We additionally used (at baseline and 32 weeks gestation) the Beck Depression Inventory (BDI, version IA; (Beck et al., 1961) and the State-Trait Anxiety Inventory (STAI; (Spielberger, 1983), two self-rated instruments measuring intensity or frequency of, respectively, depressive and anxiety symptoms. The BDI and STAI were also administered at 6-days, 2- and 12-months postnatal.

The most relevant socio-demographic and medical factors are presented in Table 1. As expected, the group with MDD in pregnancy had statistically significant higher BDI and STAIS scores both at baseline and 32 weeks gestation. No subjects were taking antidepressant medication at the baseline assessment, although 8 (16%) took antidepressants at some point during pregnancy (4 before and 4 after the baseline visit). Perhaps not surprisingly when considering the known risk factors for antenatal depression (Biaggi et al., 2016), depressed women were more likely to be unmarried or not cohabiting with a partner, to have achieved lower education, to have smoked in the index pregnancy, to be unemployed or employed at a non-professional/managerial level, or to be of black or another ethnic minority status (Table 1). Pre-pregnancy body mass index (BMI) was numerically higher in depressed women, but the difference was not statistically significant (Table 1). In order to condense the information from these socio-demographic variables, the Index of Multiple Deprivation (IMD) score was examined (Noble et al., 2004), a UK government measure of relative deprivation for small areas that covers seven aspects of deprivation. Expectedly, IMD score was significantly higher (more deprivation) in cases compared with controls (Table 1). Moreover, IMD was significantly correlated with the socio-demographic variables and with maternal ethnicity (range of r = .30–.42) but not with maternal smoking in pregnancy; hence IMD and smoking in pregnancy were both examined in analyses adjusting for potential confounders when appropriate (see data analysis, below). The groups did not differ in obstetric history or obstetric risk factors at baseline, and there were no significant group differences in medical conditions, use of medication other than antidepressant drugs, and other health indicators or health behaviors (data not shown).

**Table 1:** Characteristics of the sample at baseline and 32 weeks gestation.

	Control	Case	Statistical test and significance
	(n = 53–57)	(n = 46–49)	
Age (years), mean (SD)	32.2 (4.4)	30.7 (6.7)	t_(80.4)_ = 1.3, p = 0.20
Ethnicity, white, n (%)	43 (75.4)	24 (49.0)	**χ^2^_(1)_ = 7.9, p = 0.008**
Education, A level or higher, n (%)	53 (93.0)	31 (63.3)	**χ^2^_(1)_ = 14.1, p 0.001**
Employment status, working outside the home, n (%)	46 (80.7)	26 (53.1)	**χ^2^_(1)_ = 9.2, p = 0.003**
Classification of employment, professional or managerial, n (%)	41 (71.9)	18 (36.7)	**χ^2^_(1)_ = 13.2, p < 0.001**
Marital status, married or cohabiting, n (%)	50 (87.7)	22 (44.9)	**χ^2^_(1)_ = 22.2, p < 0.001**
IMD score, mean (SD)	28.6 (8.0)	32.2 (10.2)	**t_(87.5)_ = −2.2, p = 0.031**
Pre-pregnancy BMI, mean (SD)	*23.1* (3.8)	*24.6* (4.5)	z** = **−1.73, p = 0.08
Cigarette use in index pregnancy, n (%)	2 (3.7)	17 (35.4)	**χ^2^_(1)_ = 16.9, p < 0.001**
Antidepressant before baseline, n (%)	0	4 (8.2)	**χ^2^_(1)_ = 4.8, p = 0.043**
Antidepressant after baseline, n (%)	0	4 (8.2)	**χ^2^_(1)_ = 4.8, p = 0.043**
BDI score at baseline, mean (SD)	3.8 (2.6)	19.3 (11.4)	**t_(42.0)_ = −8.5, p = 0.001**
STAIS score at baseline, mean (SD)	27.4 (6.8)	50.8 (12.8)	**t_(57.1)_=-10.6, p = 0.001**
BDI score at 32 wks. gestation, mean (SD)	3.4 (2.6)	14.1 (10.3)	**t_(31.7)_ = −5.4, p = 0.001**
STAIS score at 32 wks. gestation, mean (SD)	29.3 (8.3)	48.3 (14.0)	**t_(47.5)_ = −6.3, p = 0.001**

Bold values signify statistical significance.

Compared with women who remained in the study, subjects who did not complete the 1 year assessment had higher BDI (9.4 ± 10.6 v 16.1 ± 10.8, z = 2.5, p = 0.013) and STAI (35.8 ± 14.9 v 47.4 ± 13.4, z = 2.7, p = 0.006) scores at baseline and a greater proportion who had smoked in pregnancy (11 (13%) v 8 (44%), χ^2^_(1)_ = 9.6, p = 0.005), which may have influenced some of the findings (see Discussion).

### 2.4 Inflammatory markers

Blood was obtained between 12 pm and 3 pm at a visit early in the third trimester (median = 27.0 weeks, range 23.9–34.9 weeks); there was no statistically significant difference in gestational age at sample acquisition between cases and controls (z = 1.1, p = 0.28). All samples were transported to the laboratory in a cooled box and processed within 2 h of venipuncture. Aliquots of serum were immediately frozen at −80 °C pending analysis. Serum high sensitivity C-reactive protein (hsCRP) was measured using an ELISA kit supplied by PZ Cormay, Poland; the assay was analyzed in batches on the Cobas Mira (intra- and inter-assay CV were 2.96% and 3.85% respectively). Serum IL-1α, IL-1β, IL-2, IL-4, Il-6, IL-8, IL-10, TNFα, vascular endothelial growth factor (VEGF), EGF, MCP-1, and INF-γ were measured using a cytokine chip array kit supplied by Randox Laboratories, UK; the kit employs a sandwich chemiluminescent immunoassay, described in our previous work (Di Nicola et al., 2013). For IL-1α, IL-4 and INF-γ, >50% of the sample was at the lowest detectable level of the assay, so these measures were not included in the subsequent analyses.

### 2.5 Salivary cortisol

*Maternal saliva samples* were obtained in the third trimester (median = 32.4 weeks, range 30.6–37.1 weeks); there was no statistically significant difference in gestational age at sample acquisition between cases and controls (z = 0.48, p = 0.63). All subjects collected two samples, using Salivettes containing a polymer swab (Sarstedt, UK), at awakening and 8 pm. Cortisol awakening response (CAR) was assessed in a subset (n = 29) that also collected samples at +15 min, +30 min, and +60 min after awakening. Subjects were given a practical demonstration, verbal and written instructions, a recording log and a mechanical timer for sample collection; emphasis was placed on the accuracy of timings and procedure, and subjects were instructed not to eat, drink or smoke in the first hour after awakening or in the thirty minutes before sample collection at 8 pm. There were no differences between cases and controls, in awakening time (7:34 h ± 0:52), the time of awakening sample collection (7:39 h ± 0:52), the interval between awakening and sample acquisition (6.70 min ± 11.35) and the time of evening sample collection (20:09 h ± 0:39).

*Infants’ saliva* samples were collected by a researcher before and 20 min after the routine immunizations at 2 and 12 months (median age = 2.1 months, range 1.62–5.54, and =12.6 months, range 11.0–18.6, respectively); there was no statistically significant difference in age for infants of cases or controls at either time point (z = 1.3, p = 0.18, and z = 0.5, p = 0.62, respectively). Infants’ saliva samples were also obtained on the following day, by the mother, at morning awakening and at 8 pm. Care was taken to avoid feeding for 15 min before a sample was taken. A Salivette and Salimetrics childrens swab (SCS) were used to collect saliva by the researcher on the immunization day; while, for ease of use, a Sorbette arrow was used for infant saliva collection the following day by the mother. As for mothers, a sample log was used to record timings and relevant information. There were no differences between infants of cases and controls, in awakening time (7:27 h ± 1:05 at 2 months and 7:19 ± 1:01 at 12 months), the interval between awakening and sample acquisition (15.6 min ± 21.0 at 2 months and 14.7 ± 21.0 at 12 months) and the time of evening sample collection (20:05 h ± 0:44 at 2 months and 19:43 ± 0:41 at 12 months).

Salivary samples were frozen at −20 °C pending analysis. Saliva cortisol was measured using a high sensitivity salivary cortisol enzyme immunoassay kit supplied by Salimetrics Europe Ltd, UK. Samples were assayed in duplicate where an adequate volume of saliva allowed. The inter-and intra-assay CV ranged from 8 to 11% and 6 to 10% respectively. The formula for the area of a trapezoid (Pruessner et al., 2003) was used to calculate (i) CAR area under the curve (AUCi) using the four samples acquired within the first hour of waking, and (ii) diurnal cortisol secretion using awakening and evening cortisol values.

### 2.6 Neonatal neurobehavioral function and infant development

The Neonatal Behavioral Assessment Scale (NBAS) (Brazelton, 1995) was used to measure neurobehavioral function in term-born babies at a median age of 6.0 days (range from 4 to 42 days); there was no statistically significant difference in age at NBAS between infants of cases or controls (z = −1.0, p = 0.30). Twenty-eight behavioral items were rated and pooled into five clusters (autonomic stability, motor, orientation, range of state and regulation of state). The Bayley Scales of Infant and Toddler Development (Bayley-III, BSID) were used to evaluate cognitive, language and motor development using a series of developmental play tasks (Bayley, 2005). BSID was used at a median age of 13.1 months (range 12.0–15.4); there was no statistically significant difference in age at BSID between infants of cases or controls (z = 0.4, p = 0.65).

### 2.7 Data analysis

The statistical analyses were performed in SPSS Statistics Version 21 (IBM Ltd, UK). The analysis plan comprised cross-sectional group comparisons of maternal antenatal biomarkers, birth outcomes, neonatal neurobehavior, infant HPA axis, and development in 1-year-olds, as well as the associations between infant factors and maternal biomarkers. For all statistical tests, the data were first examined to ensure that the assumptions of the General Linear Model (GLM) were met. In order to reduce bias, data were winsorized or log-transformed prior to analyses or the bootstrap method (with 1000 samples) was employed; raw data are presented in the figures and tables. Pearson’s chi-square (χ^2^) test of the independence of variables was used for the analysis of categorical data. Pearson’s correlation (r_p_) was used for the analysis of association between parametric continuous variables, and Spearman’s correlation (r_s_) was applied to non-parametric continuous variables. In univariate analyses, group comparisons of continuous data were made using the independent samples *t*-test. For non-parametric data, the Mann-Whitney test was used and the z score reported. Univariate analyses that showed significant differences between patients and controls were repeated after adjustment for the IMD and smoking in pregnancy if appropriate. Cohen’s δ was calculated to estimate the effect size for group differences, or effect size was expressed by partial eta squared (η_p_^2^) where ANOVA and ANCOVA were applied. Family-wise adjustment for multiple comparisons was used to identify the strongest findings. Mean and standard error of the mean are presented in graphs.

## 3 Results

### 3.1 Women with antenatal depression have increased inflammatory markers in the early 3rd trimester

Inflammatory markers (see Table 2) were compared between women with and without MDD in pregnancy at a single time point in the early 3rd trimester. Compared with controls, cases had statistically significantly higher IL-6 (δ = 0.53), IL-10 (δ = 0.53), TNFα (δ = 0.90) and VEGF (δ = 0.56) (Table 2). These differences remained significant after adjusting for IMD (p values ranging <0.001–0.041).

**Table 2:** Antenatal depression and inflammatory markers in the early 3rd trimester.

	Controls (n = 53–54) mean (SD)	Cases (n = 40–41) mean (SD)	Statistical test and significance
IL-1β (ng/l)	1.33 (1.16)	2.04 (4.94)	t_(91)_** = **−0.4, p = 0.67
IL-2 (ng/l)	1.97 (2.11)	1.94 (2.15)	t_(92)_ = 0.3, p = 0.75
IL-6 (ng/l)	0.82 (0.60)	1.64 (3.12)	**t_(68.9)_ = −2.2, p = 0.031**
IL-8 (ng/l)	2.02 (1.26)	4.95 (11.63)	t_(92)_** = **−1.4, p = 0.18
IL-10 (ng/l)	0.66 (0.62)	1.55 (5.17)	**t_(63.0)_ = −2.1, p = 0.043**
TNFα (ng/l)	1.10 (0.88)	1.59 (0.99)	**t_(60.1)_ = −3.5, p = 0.003**
VEGF (ng/l)	3.20 (3.46)	6.47 (12.58)	**t_(92)_ = −2.7, p = 0.008**
EGF (ng/l)	16.07 (23.85)	21.12 (26.82)	t_(58.9)_** = **−1.8, p = 0.70
MCP-1 (ng/l)	51.56 (38.21)	67.66 (60.29)	t_(67.3)_** = **−0.8, p = 0.40
hsCRP (mg/l)	5.31 (6.83)	5.14 (6.41)	t_(93)_ = 0.8, p = 0.43

Bold values signify statistical significance.

Potential confounders did not affect these results. Smoking was not associated with any of the inflammatory markers. Moreover, even though pre-pregnancy BMI was not statistically different between cases and controls, we explored its possible association with inflammatory biomarkers: BMI was negatively associated with IL-10 only; inclusion of BMI in the covariate model for IL-10 did not change the findings. It is important to highlight that only TNFα remained statistically significant after adjustment for multiple comparisons, and thus should be considered the most robust finding among the inflammatory biomarkers. There was no statistically significant difference between cases and controls in IL-1β, IL-2, IL-8, EGF, MCP-1 or hsCRP (Table 2).

Finally, as documented in section 2.2, of the 49 cases who had been depressed in pregnancy up to the baseline assessment, 31 (63%) cases met DSM-IV criteria for MDD at the time of sample collection and 18 (37%) cases no longer met criteria; however, t-tests revealed no statistically significant difference in the inflammatory markers between cases who did and did not meet criteria for MDD at sample acquisition (p values ranging 0.11–0.47), other than for IL-10, which was higher in cases who did not meet criteria for depression at baseline than cases who did (0.83 ng/l ± 0.39 vs. 0.57 ng/l ± 0.29 respectively, t_(39)_ = −2.45, p = 0.019). These secondary analyses confirm the notion that depressed pregnant women maintain a pro-inflammatory signature at the end of the second trimester even if their depression has improved by then.

### 3.2 Women with antenatal depression have increased diurnal cortisol secretion, and blunted cortisol awakening response, in the 3rd trimester

**Fig. 1:**
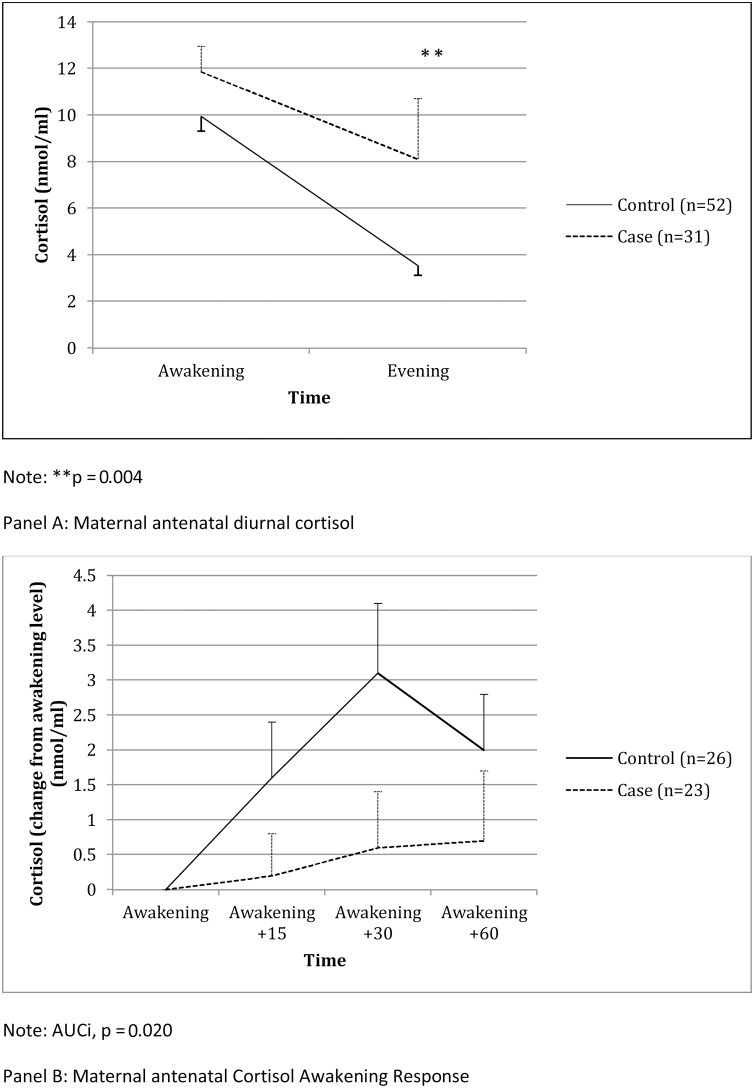
Antenatal depression and maternal hypothalamic-pituitary axis at 32 weeks gestation.

Basal HPA axis activity was compared between women with and without MDD in the 3rd trimester. Depressed women had a higher diurnal cortisol secretion (AUC, nmol/ml/minute; t_(42.8)_ = −2.9, *p* = 0.006, δ = 0.89; p = 0.001 after adjustment for IMD) (Fig. 1, Panel A). Moreover, in terms of individual time-points, depressed women had higher awakening cortisol levels (t_(44.1)_ = −1.8, p = 0.07, δ = 0.54; p = 0. 027 after adjustment for IMD), and significantly higher evening cortisol levels (t_(81)_ = −2.9, p = 0.004, δ = 0.64; p = 0.006 after adjustment for IMD). Lastly, depressed women had a statistically significant smaller (blunted) CAR (AUCi, nmol/ml/minute; (t_(47)_ = 2.4, p = 0.020, δ = 0.70; p = 0.016 after adjustment for IMD) (Fig. 1, Panel B).

Potential confounders did not affect these results. Smoking was not associated with these cortisol measures. Again, although pre-pregnancy BMI was not statistically different between cases and controls, we explored its possible association with the HPA axis measures; it was negatively associated with CAR only; inclusion of BMI in the covariate model for CAR did not change the findings. Diurnal and evening cortisol remained statistically significant even after adjusting for multiple comparisons. Finally, t-tests revealed no statistically significant difference in the HPA axis measures between cases who did and did not meet criteria for MDD at the baseline visit (p values ranging 0.25-0.95).

### 3.3 Women with antenatal depression have babies with lower gestational age at birth

Gestational age at birth for women with spontaneous onset of labor was lower in babies of cases compared with those of controls, by approximately 8 days (mean weeks 39.2 ± 2.6 v 40.4 ± 1.4, t_(69)_ = 2.9, p = 0.005, δ = 0.70; p = 0.012 after adjustment for IMD), and smoking was not associated with this measure. There was no statistically significant difference between cases and controls in any other obstetric outcome or obstetric detail (Supplementary Material: Table S1).

### 3.4 Neonates who had been exposed to antenatal depression have suboptimal neurobehavioral function at 6 days postnatal

The neurobehavioral assessment (NBAS) of full-term babies conducted at 6 days postnatal showed that babies exposed to antenatal depression demonstrated poorer performance on all five clusters: autonomic stability (δ = 0.85), regulation of state (δ = 0.61), range of state (δ = 0.53), orientation (δ = 1.22) and motor (δ = 0.45) (Table 3).

**Table 3:** Antenatal depression and neonatal neurobehavioral examination (NBAS).

	Infants of controls	Infants of cases	Test & statistical significance
	(n = 56), mean (SD)	(n = 43), mean (SD)	
Autonomic stability	6.17 (1.13)	5.26 (0.99)	**t_(97)_ = 4.2, p < 0.001**
Regulation of state	6.39 (1.28)	5.52 (1.63)	**t_(97)_ = 3.0, p = 0.004**
Range of state	3.30 (0.81)	3.66 (0.80)	**t_(97)_ = −2.6, p = 0.013**
Orientation	7.49 (1.26)	6.47 (1.45)	**t_(63.9)_ = 4.9, p = 0.001**
Motor	5.66 (0.70)	5.33 (0.81)	**t_(97)_ = 2.1, p = 0.049**

Bold values signify statistical significance.

The analyses were adjusted for IMD and other potential confounders (sociodemographic, health indicators, pregnancy, delivery or neonatal) that differed between cases and controls, including gestational age at birth and smoking in pregnancy. For all the NBAS clusters, except ‘range of state,’ the effect of depression remained significant after adjustment for IMD and these other potential confounders (p values ranging 0.001-0.036). For ‘range of state’ there was a significant effect of smoking in pregnancy (p = 0.030) and the effect of depression was no longer significant after adjusting for this variable. Autonomic stability, regulation of state and orientation remained statistically significant even after adjusting for multiple comparisons.

### 3.5 Infants who had been exposed to antenatal depression have normal cortisol function at 2 months of age

Cortisol function of the two-month-old infants was assessed by measuring cortisol before and 20 min after immunizations, as well as morning and evening levels.

**Fig. 2:**
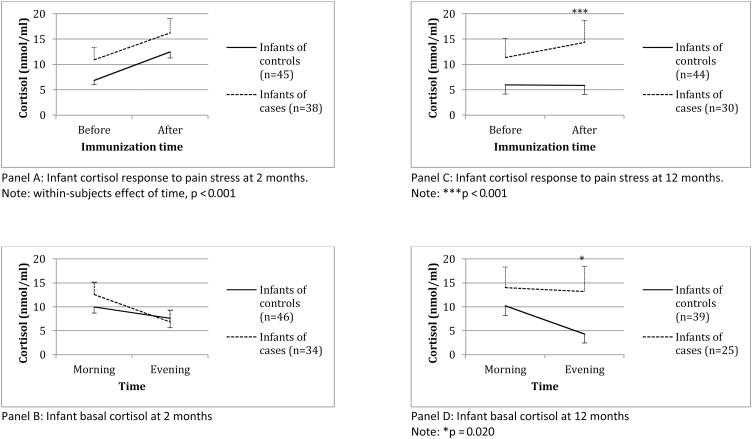
Infant cortisol at 2 and 12 months.

For cortisol reactivity to stress (Fig. 2, Panel A), the mixed design ANOVA revealed a statistically significant within-subjects effect of time on cortisol (F_(1, 81)_ = 27.0, p < 0.001, η_p_^2^ = .25), that is, cortisol increased following immunization in both depression-exposed and non-exposed infants. However, there was no interaction between caseness and time (F_(_1,81_)_ = 0.4, p = 0.51, η_p_^2^ = .01) and no between-subjects effects (F_(1, 81)_ = 0.6, p = 0.45, η_p_^2^ = .01). Thus, the magnitude of the cortisol response did not differ between those infants who had and had not been exposed to antenatal depression.

Basal HPA axis activity in the eight-week-old infants was assessed the day after their immunization by measuring awakening and evening cortisol (mean time 07:47 h ± 1:08 and 20:05 h ± 0:45` respectively; there was no difference in the time of sample acquisition between cases and controls). There was no statistically significant difference in either measure between the exposed and non-exposed infants (p values 0.70 and 0.46 respectively) (Fig. 2, Panel B).

### 3.6 Infants who had been exposed to antenatal depression have hyperactive cortisol responses at 12 months of age

Cortisol function of the 12-month-old infants was assessed, as at 2 months, by measuring cortisol before and 20 min after the immunizations, as well as morning and evening levels.

For cortisol reactivity to stress (Fig. 2, Panel C), mixed design ANOVA demonstrated a statistically significant between-subjects effect of caseness on cortisol levels (F_(1, 72)_ = 0.5, p = 0.002, η_p_^2^ = .13), as well as a statistical trend for an interaction between caseness and time (F_(1, 72)_ = 3.4, p = 0.07, η_p_^2^ = 0.05), but no within-subjects effect (F_(1, 72)_ = 1.9, p = 0.17, η_p_^2^ = 0.03). These statistical findings were driven by an increase in cortisol levels following stress in the infants exposed to antenatal depression, but not in the non-exposed infants (Fig. 2, Panel C). In fact, infants exposed to antenatal depression had higher cortisol levels after the immunization compared with control infants (t_(72)_ = −3.7, p < 0.001, δ = 0.87) but not before (t_(72)_ = −1.6, p = 0.11, δ = 0.38). The mixed design ANOVA used to measure the cortisol response to stress takes account of the multiple testing, and thus this group difference is statistically robust.

Given the well-established continuity between maternal antenatal and postnatal depressive symptoms (Plant et al., 2017), in addition to the potential confounders of IMD, smoking in pregnancy and gestational age at birth, we also examined the effect of maternal postnatal depressive symptoms (BDI) on these findings. We included maternal depressive symptoms at 12 months (at the same time as the cortisol assessment) as well as the total postnatal burden of maternal depressive symptoms (the average BDI score at 6 days and at 2 and 12 months). As expected, compared with control women, women with antenatal depression had higher BDI score both at 12 months postnatal (9.7 ± 9.2 vs. 3.3 ± 3.4, t_(29.4)_ = −3.4, p = 0.002, δ = 1.25) and as total postnatal burden (9.9 ± 7.6 vs. 3.5 ± 2. 9, t_(33.2)_ = −4.6, p < 0.001, δ = 1.60). Only average postnatal symptoms of depression were associated with the infant cortisol, therefore average postnatal BDI and IMD were included in the ANOVAS. The between-subjects effect of caseness in the mixed design ANOVA remained significant (p = 0.024), as did the difference between exposed and non-exposed infants in the post-immunization cortisol (p = 0.021). Thus, the increase in cortisol levels following stress in the infants exposed to antenatal depression was not driven by the postnatal depressive symptoms or other potential confounding factors.

Lastly, as at 2 months, basal cortisol activity in the 12-month-old infants was assessed the day after their immunization by measuring awakening and evening cortisol levels (mean time 07:37 h ± 0:57, and 19:42 h ± 0:40, respectively; there was no difference in the times of sample acquisition between cases and controls). Infants exposed to antenatal depression had higher evening cortisol at 12 months (t_(62)_ = −2.4, p = 0.020, δ = 0.61), but not morning cortisol, (t_(62)_ = −0.7, p = 0.49, δ = 0.18 (Fig. 2, Panel D). As for cortisol stress-response, we examined the effect of IMD, smoking in pregnancy, gestational age at birth and concurrent and total postnatal burden of maternal postnatal depressive symptoms. Again, only average postnatal symptoms of depression were associated with infant cortisol, therefore average postnatal BDI and IMD were included in the ANOVAS. The difference in evening cortisol became non-significant after adjustment for IMD and average postnatal BDI (p = 0.44). Therefore, the higher evening cortisol levels in infants exposed to antenatal depression were driven by the socio-demographic factors and by maternal postnatal depressive symptoms.

### 3.7 Infants who had been exposed to antenatal depression have normal development at 12 months postnatal

Compared with infants of healthy women (n = 50), those exposed to depression in utero (n = 35) did not differ in cognitive (114.3 ± 14.0 vs. 109.6 ± 14.0, t_(83)_ = 1.5, p = 0.11), language (100.9 ± 12.4 vs. 98.6 ± 12.3, t_(83)_ = 1.4, p = 0.16) or motor (101.4 ± 10.4 vs. 103.6 ± 12.0, t_(83)_ = −0.9, p = 0.38) development at 12 months of age, as measured by BSID. The findings are the same both for the entire sample and solely for full-term infants.

### 3.8 Associations between maternal antenatal stress-related biology and infant measures

Correlations were used to examine the associations between maternal antenatal measures that differed between cases and controls (i.e., inflammatory markers and cortisol levels) and the infant measures that differed between cases and controls and were uniquely explained by the antenatal exposure (i.e., gestational age at birth, NBAS, and post-stress cortisol at 12 months) (Table 4).

**Table 4:** Correlations for maternal antenatal and infant measures.

	Gestational age at birth	NBAS Autonomic stability	NBAS Regulation of state	NBAS Orientation	NBAS Motor	1 year-old infant’s cortisol after the immunizations
Diurnal cortisol	0.05	−0.14	0.03	−0.08	−0.01	0.28*
Evening cortisol	−0.14	−0.02	−0.1	−0.12	0.11	0.32**
CAR (AUCi)	0.17	0.04	−0.11	0.1	0.04	−0.29
IL-6	−0.21	−0.02	−0.18	−0.08	0.15	0.15
IL-10	−0.14	−0.07	−0.28**	−0.05	0.04	0.35*
TNFα	−0.12	−0.22*	−0.11	−0.18	−0.06	0.37**
VEGF	−0.08	−0.23*	−0.09	−0.22*	0	0.47***

Note. Correlation coefficient - Spearman’s r.

Bold values signify statistical significance.

* p ≤ 0.05.

** p ≤ 0.01.

*** p ≤ 0.001.

There were no correlations between maternal antenatal biomarkers and gestational age at birth. However, there were statistically significant correlations between maternal biomarkers (TNFα, VEGF, IL-10 and diurnal cortisol) and the NBAS scores of full-term babies (autonomic stability, regulation of state, and orientation; range of all r = 0.22–0.28, all p values <0.05). These correlations were all in the expected direction, i.e., elevated biomarker levels correlated with less optimal neurobehavioral function. There were also statistically significant correlations between maternal biomarkers (TNFα, VEGF, IL-10 and evening cortisol) and infant cortisol after the immunizations at 12 months (all r ranging from 0.28 to 0.47, all p values <0.022). Again, these correlations were all in the expected directions, i.e., elevated maternal antenatal biomarker levels correlated with higher infant cortisol. Taken all together, these correlations support the notion that neonatal neurobehavioral function at 6 days and cortisol stress response at 12 months are an embedded biological programming effect that is associated with the antenatal biological changes found in women with MDD in pregnancy.

## 4 Discussion

We used a prospective longitudinal design and demonstrated that women with clinically-significant antenatal MDD have: (i) abnormal stress-related biology (inflammation and cortisol) in pregnancy; (ii) shorter length of gestation; (iii) neonates with sub-optimal neurobehavioral function; and (iv) one-year-old infants with increased cortisol reactivity to stress. Our findings are in keeping with the hypothesis that maternal antenatal MDD has a programming effect on offspring neurobehavioral function and HPA axis activity, and, furthermore, that maternal antenatal inflammation and cortisol are involved in the mechanistic pathway of these programming effects.

### 4.1 Maternal antenatal stress-related biology

Compared with healthy women, those with antenatal MDD have higher levels of 3^rd^ trimester inflammatory markers (IL-6, IL-10, VEGF and TNFα), HPA axis overactivity as indexed by raised evening cortisol and increased total daily cortisol secretion, and a blunted cortisol awakening response. Our findings are broadly consistent with previous literature, although the findings from previous studies of antenatal depression and inflammation or HPA axis appear mixed, and they are difficult to compare as they use different definitions or measures of depression and different measurements of inflammation or HPA axis, at different stages of gestation, different times of day and in different tissues. The most-studied inflammatory markers are CRP, IL-1b, IL-6 and TNFα, which are all more commonly raised in relation to increased depressive symptomatology (Scrandis et al., 2008; Christian et al., 2009; Cassidy-Bushrow et al., 2012; Azar and Mercer, 2013; Haeri et al., 2013), than lowered (Shelton et al., 2015; Edvinsson et al., 2017); however, these same studies also have null findings for some of these inflammatory markers.

The majority of studies of the HPA axis and operationally defined depression have demonstrated higher saliva cortisol (Evans et al., 2008; Monk et al., 2011; O’Keane et al., 2011), and studies comparing pregnant women with high or low levels of self-rated symptoms of depression largely (Lundy et al., 1999; Field et al., 2001, 2004b; Parcells, 2010; Peer et al., 2013), but not always (Shea et al., 2007; Braithwaite et al., 2016), report increased cortisol. In general, for the HPA axis the findings appear to be more robust when depression is present at disease level. In contrast with our findings, two studies using diagnostic interviews have shown no effect of antenatal depression on CAR (Hellgren et al., 2013; O’Connor et al., 2013b). However, both studies were broader in their inclusion of not only major depression, but also minor depression and/or depression ‘not otherwise specified’ (NOS) or defined as high scores on EPDS. Furthermore, although O’Connor et al. (2013b) measured CAR at a similar length of gestation as our study, Hellgren et al. (2013) measured CAR relatively late in pregnancy, at 36–39 weeks gestation; thus, overall these studies are not directly comparable to ours. Animal studies of prenatal stress (PNS) mirror our findings: for example, corticosterone is elevated in pregnant rats subjected to electric tail shock or restraint stress (Ward and Weisz, 1984; Weinstock et al., 1988; Takahashi et al., 1998; Williams et al., 1999) and in pregnant rhesus monkeys subjected to noise stress (Schneider et al., 1999).

Maternal antenatal HPA axis is critical in pregnancy physiology, e.g., fetal maturation and timing of parturition. Consistent with this, and with previous research, depressed women in our study do indeed have shorter length of gestation. Previous meta-analyses have also shown an association between antenatal depression and preterm birth (Grote et al., 2010; Grigoriadis et al., 2013), although the evidence for shortened length of gestation per se is mixed (Van Dijk et al., 2010; O’Keane et al., 2011; Grigoriadis et al., 2013). Interestingly, we find no significant correlations between maternal inflammatory biomarkers and shorter length of gestation, while two previous studies did: Blair et al. (2015) found a negative correlation between IL-8 (at 19–30 weeks gestation) and length of gestation, although this finding was only apparent in African American (n = 79), not European American women; and Coussons-Read et al. (2012) found a negative correlation between both IL-6 and TNFα (at 28–30 weeks gestation) and length of gestation in 173 mostly Hispanic women, who were free from psychiatric disorder. We found a statistical trend for a negative correlation between IL-6 and gestational age, while the other correlations were in the same direction but not statistically significant (Table 4). The clearer findings in these two aforementioned papers might be explained by the different ethnicity of the populations under study as well as possibly (for Coussons-Read et al., 2012) by a larger statistical power. Regarding previous studies of maternal HPA axis activity and length of gestation, the most robust finding is the relationship between preterm birth and raised CRH (which was not examined in this study) (McLean et al., 1995; Wadhwa et al., 1998; Sandman et al., 2006).

### 4.2 Infant cortisol stress reactivity

The principal focus of this study was to examine the developmental programming effects of in utero exposure to MDD on offspring cortisol reactivity to the stress of immunization. We found that, compared with infants of healthy women, those exposed to MDD in utero show a larger cortisol stress response at 12, but not at 2 months. This increased post-stress cortisol persists when symptoms of postnatal maternal depression are taken into account, indicating a biologically embedded antenatal effect. To our knowledge, this is the first prospective study linking operationally defined antenatal MDD and cortisol response to stress in 1-year-old offspring, and our findings extend and confirm previous studies of PNS and infant stress response both in humans and in animals (Weinstock, 2008). For example, high levels of antenatal symptoms of depression in a non-clinical sample were associated with a larger cortisol response to immunization in infants (Fernandes et al., 2015). Similarly, higher levels of maternal daily hassles and pregnancy-related anxiety were associated with higher cortisol in the 30 min after immunization in four- to six-year-old children (Gutteling et al., 2004). However, antenatal perceived stress predicted greater cortisol reactivity to heelstick in neonates in one study (Leung et al., 2010) but not another (Davis et al., 2011a): these contrasting findings may be explained by retrospectively-rated stress in the first but not the second study. In animals, we and others have shown that adult rats born from dams exposed to restraint stress in the last week of gestation show HPA axis hyperactivity, depressive-like behavior, and changes in hippocampal gene expression that are overlapping to those found in the blood of depressed patients (Anacker et al., 2013a, b; Cattaneo et al., 2018).

Interestingly, previous research has shown that a cortisol stress response is apparent at birth, but that it diminishes with age, such that by 12 months there is little or no cortisol response to pain (Jansen et al., 2010). This is the first study to show that, in contrast to the typically expected lack of cortisol response to pain stress in 12-month-olds, the response persists in those infants who had been exposed to MDD in utero. In contrast, at 2 months there was a comparable increase in cortisol in both cases and controls. This demonstrates that the effect of antenatal MDD in utero is not apparent at an early postnatal stage but it does become apparent later, at a developmental stage when healthy infants generally no longer exhibit a cortisol stress response.

### 4.3 Associations between maternal stress-related biology and infant cortisol

In our study, infant cortisol after the immunization at 12 months is significantly, positively correlated with maternal antenatal inflammatory markers (IL-10, TNFα and VEGF) and evening cortisol. These correlations are in the same direction as hypothesized, i.e. women with antenatal MDD have higher IL-10, TNFα, VEGF and evening cortisol in pregnancy, and their infants have higher cortisol after their immunizations. This association supports the hypothesis of a mechanistic link between the antenatal biological environment and offspring cortisol stress regulation. Moreover, these findings extend those of other studies in humans that demonstrate an association between maternal antenatal cortisol (Gutteling et al., 2004, 2005; Davis et al., 2011a), or amniotic fluid cortisol (O’Connor et al., 2013a), and infant cortisol stress response. Further supporting these findings, research in rodents has shown that an immune challenge during pregnancy augments the offspring HPA axis stress-response (Reul et al., 1994; Samuelsson et al., 2004). Moreover, animal studies using adrenalectomized pregnant rodents have provided a definitive demonstration that maternal glucocorticoids mediate the long-term effects of prenatal stress on offspring HPA axis activity (Barbazanges et al., 1996; Maccari et al., 2003; Wilcoxon and Redei, 2007). Interestingly, the notion that antenatal depression *specifically* programs cortisol stress reactivity through maternal inflammation and cortisol functions is further supported by our finding that the evening cortisol of 12-month infants, a measure of resting HPA axis, is linked to *postnatal* maternal depression.

### 4.4 Offspring neurobehavioral function and development

Compared with neonates of healthy women, those exposed to MDD in utero show suboptimal neurobehavioral function at 6 days, indexed by NBAS examination; however, there is no group difference in cognitive, language or motor development at 12-months of age. The findings in neonates mirror previous studies of antenatal depression and offspring neurobehavioral function, as described in the introduction (Field et al., 2004a; Diego et al., 2005; Goodman et al., 2011; Pacheco and Figueiredo, 2012), and further demonstrate that the adverse effect of MDD in pregnancy is not limited to pregnancy biology and its immediate sequelae, but extends to affect the offspring. Of note, only one NBAS cluster, ‘range of state’, seems to be influenced more by maternal smoking and mode of delivery than by antenatal depression per se. ‘Range of State’ measures infant arousal, lability and irritability; and previous studies of NBAS and smoking in pregnancy are in keeping with the finding of an effect of smoking on irritability, although these studies did not control for maternal mood (Oyemade et al., 1994; Mansi et al., 2007; Hernandez-Martinez et al., 2012). Likewise, the above-mentioned studies of antenatal depression and NBAS did not control for maternal smoking. Our study is the first to demonstrate that some neurobehavioral abnormalities in infants exposed to antenatal depression are directly related to the in utero biology (see below) while others appear to be related to smoking in pregnancy.

As mentioned above, some NBAS clusters are significantly correlated with maternal antenatal inflammatory and HPA axis markers. These correlations are in the same direction as hypothesized, i.e., elevated maternal antenatal stress-related markers are associated with less optimal NBAS scores, again supporting the hypothesis of a mechanistic link for these neurobehavioral outcomes. Other studies have demonstrated associations between maternal antenatal inflammation and offspring behavior; for example, a study has shown that IL-6 is associated with impulse control in 2-year-old offspring (Graham et al., 2017). Moreover, maternal antenatal HPA axis has also been previously associated with offspring behaviors, such as crying and fussing in babies from one to seven weeks of age (de Weerth et al., 2003) or negative reactivity at 2 months (Davis et al., 2005, 2007). However, these studies have not examined this association within the context of clinically significant depression. Animal evidence also supports our finding; for example, maternal antenatal immune activation in mice has long-term effects on offspring behavior, such as deficient social and communicative behavior (Malkova et al., 2012), and on brain structure and histopathology, such as altered pyramidal and nonpyramidal cell density (Fatemi et al., 2002); and offspring of rats given synthetic glucocorticoids in pregnancy display increased depressive and anxious behavior (Oliveira et al., 2006).

The lack of effect of antenatal depression on developmental outcomes at 12 months is potentially surprising. However, there are limited data in humans on the effects of depression in pregnancy on offspring developmental outcome, and results are conflicting (Waters et al., 2014). Some large studies of offspring of women with a high level of symptoms of depression in pregnancy (>1000 of children, ranging from 18-months to 8-year-olds) have found developmental delay, decreased cognitive development, and lower IQ (Deave et al., 2008; Tse et al., 2010; Evans et al., 2012). Likewise, a study of >200 18-month-old children found an association between high levels of symptoms of depression and lower cognitive development (Koutra et al., 2013). However, another study of >300 children using the same scale as our study and administered at the same time-point (the BSID at 12 months) found no such relationship (Bandoli et al., 2016). In the current study we found that both cognitive and language scores were numerically lower in infants of depressed mothers, although the differences did not reach statistical significance. Thus, the lack of finding may have been an issue of power, or alternatively due to the developmental stage of the children. Clarification of the effect of antenatal depression on infant development requires further follow-up study in these children. As discussed below, it is possible that the lack of difference in the BSID scores may be partly due to the analyses being limited to a less severely depressed sample.

### 4.5 Limitations

Despite a number of important strengths, including the prospective design, diagnostic assessment by semi-structured interview, and a broad assessment of inflammation and HPA axis markers in women and offspring over time, there are some limitations. First, subjects were drawn from a population with broad diversity in socio-economic status and ethnicity; although we have controlled for social deprivation (IMD), the numbers were too small to complete any analyses in specific social or ethnic subgroups. Furthermore, subjects were medically healthier compared with a general pregnant population, due to the exclusion criteria for the study, and indeed their pre-pregnancy BMI was not associated with depression. Second, the majority of cases had a previous history of depression or of other (non-psychotic) mental disorders, and we cannot exclude a genetic component linking maternal psychopathology with offspring biological or behavioral changes; nevertheless, we have been able to show that our main finding – the increase offspring cortisol response - is driven by depression in pregnancy and not by depression in the postpartum, thus indicating that specific effects are present for depression in pregnancy, over and above any generic risk associated with depression at other times. Third, although cases were free from antidepressant medication at baseline, 8 subjects took antidepressants later in pregnancy. These effects could not be quantified because of the infrequency of this potential confounding factor; however, exclusion of these eights subjects from the analyses did not change the findings (data not shown). Forth, principally because of difficulties recruiting such an unwell group of pregnant women, the sample size is relatively small. This limitation is compounded by sample attrition over the 15 months of study, and those who dropped out had higher BDI and STAI scores at baseline; thus, perhaps understandably, those who had more severe psychopathology early in pregnancy then seem to have struggled to remain involved in the study. Overall, these findings do not detract from our main biological finding in infants (the increased cortisol response at 12 months), as these new data show that this effect is present also in the potentially less severe group of women and their infants who had the follow-up visit. However, as mentioned above, it is possible that the lack of difference in the BSID scores may be partly due to the analyses being limited in this less severe sample. Fifth, undoubtedly, the study’s impact comes from the coexisting measures of both biological and clinical variables from both mothers and infants, over pregnancy and the first postnatal year. However, this brings the potential confounder of multiple comparisons. We have therefore conducted family-wise adjustment, and identified that the differences in maternal TNFα and diurnal and evening cortisol levels, and in infants’ autonomic stability, regulation of state, orientation as well as cortisol response to stress at 12 months, as the most statistically robust findings. Lastly, it is essential to emphasize that the proposed causative biological pathways, linking maternal cortisol and inflammatory biomarkers with infants’ NBAS scores and cortisol response to stress, are only supported by correlational analyses. Although these correlations are all in the expected direction, i.e., elevated maternal biomarker levels correlating with less optimal neurobehavioral function and higher cortisol response in infants, only testing for mediation would be able to unequivocally confirm these causal pathways. This, however, is inadvisable in our study due to the limited sample size. As such, these associations should considered at best suggestive, and requiring further validation and replication in larger samples.

**In conclusion** the current study has addressed an existing gap in the literature regarding the possible programming effects of operationally defined, clinically significant antenatal depression, and represents the first step towards understanding the convergent inflammation- and cortisol-related mechanisms for developmental programming in humans. Studying the effects of depression at a disease level ensures that stress is meaningful and produces clinically significant impairment. Most importantly, antenatal depression is common (Gavin et al., 2005) and is associated with adverse obstetric outcomes (Grote et al., 2010; Grigoriadis et al., 2013), but is also readily diagnosed and treated, thus providing an opportunity for intervention (Pariante, 2015), ultimately preventing the transmission of abnormal stress biology, and related psychopathology, to the next generation.

## Funding and disclosure

The work was supported by the following grants: The UK National Institute for Health Research (NIHR) Biomedical Research Centre at the South London and Maudsley NHS Foundation Trust and King’s College London (CMP, SO); the UK Medical Research Council (MR/L014815/1 and MR/J002739/1) (CMP); The Lullaby Trust (formerly known as the Foundation for the Study of Infant Deaths) (263) (CMP, SC, SO); the Psychiatry Research Trust (CMP, SO); the Brain and Behavior Research Foundation (SO); and Singapore Health Services Health Manpower Development Plan Award (2012) (TEC). The funding sources played no role in the study design, writing, or analysis of the paper and results.

## Declaration of interest statement

CMP has received research funding from pharmaceutical companies interested in the development of new antidepressants, such as Johnson & Johnson and Eleusis Ltd, but this project is unrelated to this funding; there are no further declarations of interest.
