# Corrigendum to “Antenatal depression programs cortisol stress reactivity in offspring through increased maternal inflammation and cortisol in pregnancy: The Psychiatry Research and Motherhood – Depression (PRAM-D) study” [Psychoneuroendocrinology 98 (2018) 211–221]

**DOI:** 10.1016/j.psyneuen.2020.104795

**Published:** 2020-09

**Authors:** S. Osborne, A. Biaggi, T.E. Chua, A. Du Preez, K. Hazelgrove, N. Nikkheslat, G. Previti, P.A. Zunszain, S. Conroy, C.M. Pariante

**Affiliations:** a King’s College London, Institute of Psychiatry, Psychology and Neuroscience, Department of Psychological Medicine, Section of Perinatal Psychiatry & Stress, Psychiatry and Immunology, The Maurice Wohl Clinical Neuroscience Institute, Cutcombe Road, London, SE5 9RX, UK; b King’s College London, Institute of Psychiatry, Psychology and Neuroscience, Department of Psychological Medicine, Section of Psychosis Studies, London, SE5 9AF, UK; c Department of Psychological Medicine, KK Women’s and Children’s Hospital, 100 Bukit Timah Road, Singapore 229899, Singapore; d King’s College London, Institute of Psychiatry, Psychology and Neuroscience, Department of Basic and Clinical Neuroscience, The Maurice Wohl Clinical Neuroscience Institute, Cutcombe Road, London, SE5 9RX, UK; e Department of Mental Health and Addiction, Via Risorgimento 57 42123, Reggio Emilia, Italy

The authors regret that an error was found in the syntax used to create the variable for the NBAS ‘Range of State’ cluster. They believe this came about because of a mistake in the 2011 version of the NBAS manual (Brazelton and Nugent, 2011). According to the Cluster Soring Criteria in the 2011 manual, the raw score should be used for ‘Rapidity of Build-up’ (one of the four items that makes up the ‘Range of State’ cluster). However, the manual was issued with an erratum stating that ‘Rapidity of Build-up’ should in fact be recoded as per the 1995 manual (Brazelton and Nugent, 1995). This, unfortunately, seems to have been missed when the syntax was created.

Using the correctly recoded item there is no longer a significant difference between babies born to controls and those born to cases in range of state scores.

The authors would, therefore, like to make the following changes to the manuscript:

**Results Paragraph 3.4.** ‘***Neonates who had been exposed to antenatal depression have suboptimal neurobehavioral function at 6 days postnatal’***. N**ow reads:**

The neurobehavioral assessment (NBAS) of full-term babies conducted at 6 days postnatal showed that babies exposed to antenatal depression demonstrated poorer performance in four of the five clusters: autonomic stability (δ = 0.85), regulation of state (δ = 0.61), orientation (δ = 1.22) and motor (δ = 0.45). There was no statistically significant difference between exposed and non-exposed babies in range of state scores (Table 3).

For the four NBAS clusters on which the babies differed, the analyses were adjusted for the Index of Multiple Deprivation (IMD) and other potential confounders (sociodemographic, health indicators, pregnancy, delivery or neonatal) that differed between cases and controls, including gestational age at birth and smoking in pregnancy. For all four of the NBAS clusters, the effect of depression remained significant after adjustment for IMD and these other potential confounders (p values ranging 0.001−0.036). Autonomic stability, regulation of state and orientation remained statistically significant even after adjusting for multiple comparisons.

**Table 3, row 3. Now reads:**

**Table:** 

	Infants of controls (n = 56), mean (SD)	Infants of cases (n = 43), mean (SD)	Test & statistical significance
Range of State	3.18 (0.85)	3.28 (0.75)	t_(97)_ = −0.7, p = 0.51

The discussion of this finding (Section 4.4, 1^st^ Paragraph, from “Of note, only one cluster…” to the end of the paragraph) should also no longer be considered relevant.

