# Supplementary material for: Antenatal depression programs cortisol stress reactivity in offspring through increased maternal inflammation and cortisol in pregnancy: The Psychiatry Research and Motherhood – Depression (PRAM-D) Study
Source: Psychoneuroendocrinology. 2018 Dec;98:211–21. doi: 10.1016/j.psyneuen.2018.06.017 (PMC6215770; doi:10.1016/j.psyneuen.2018.06.017)
Supplement: Supplementary file 1 [file mmc1.docx]

Table S1 Obstetric outcomes and obstetric details

|  | **Controls (n=52-57)** | **Cases (n=46-49)** | **Statistical test and significance** |
| --- | --- | --- | --- |
| **Spontaneous onset of labour, n (%)**  **Mode of delivery, vaginal, n (%)**  **Use of pharmacological analgesia, n (%)**  **Duration of ROM (hours), mean (SD)**  **Duration of labour (hours), mean (SD)^[[1]](#footnote-1)^**  **Sex of the baby, male, n (%)**  **APGAR scores at 1 minute, mean (SD)**  **APGAR scores at 5 minutes, mean (SD)**  **Preterm birth, n (%)**  **Low birth weight baby, n (%)**  **Small for gestational age (SGA), n (%)**  **Large for gestational age (LGA), n (%)**  **Birth weight (g), mean (SD)**  **Head circumference (HC) (cm), mean (SD)^[[2]](#footnote-2)^**  **HC controlling for GA at birth, mean (SD)^[[3]](#footnote-3)^** | 40 (71.4)  43 (75.4)  45 (86.5)  7:39 (14:01)  7:06 (4:46)  32 (56.1)  8.59 (1.49)  9.66 (0.64)  1 (1.8)  1 (1.8)  3 (5.4)  9 (16.1)  3512 (428)  34.90 (1.26)  34.77 (0.18) | 32 (68.1)  36 (73.5)  36 (78.3)  5:33 (11:09)  7:03 (7:37)  27 (55.1)  8.83 (0.90)  9.72 (0.46)  3 (6.1)  3 (6.5)  4 (8.7)  5 (10.9)  3380 (540)  34.09 (1.44)  34.27 (0.21) | χ^2^_(1)_=0.1, p=0.83  χ^2^_(1)_=0.0, p=0.83  χ^2^_(1)_=1.2, p=0.30  z=-1.5, p=0.14  z=-1.2, p=0.21  χ^2^_(1)_=0.0, p=1.0  z=-0.1, p=0.94  z=-0.0, p=1.0  χ^2^_(1)_=1.4, p=0.33  χ^2^_(1)_=1.5, p=0.33  χ^2^_(1)_=0.4, p=0.70  χ^2^_(1)_=0.6, p=0.57  z=-1.0, p=0.32  **t_(83)_=2.7, p=0.012**  F_(1, 82)_=3.2, p=0.08 |

1. Not all subjects had a labour, therefore the data presented is for controls, n = 47 and cases, n = 37. [↑](#footnote-ref-1)
2. Not ascertained on the full sample (controls n = 49 and cases n = 36). [↑](#footnote-ref-2)
3. Head circumference evaluated at a gestational age at birth of 40.25 weeks. [↑](#footnote-ref-3)
